# Interrupted time series design to evaluate ICD-9-CM to ICD-10-CM coding changes on trends in Colorado emergency department visits related to traumatic brain injury

**DOI:** 10.1186/s40621-021-00308-y

**Published:** 2021-04-19

**Authors:** Lauren Alexis De Crescenzo, Barbara Alison Gabella, Jewell Johnson

**Affiliations:** 1 Colorado School of Public Health, Denver, CO USA; 2 Colorado Department of Public Health and Environment, 4300 Cherry Creek Drive South, A4, Denver, CO USA

**Keywords:** Traumatic brain injury, Hospital emergency department, Regression analysis, International classification of disease codes

## Abstract

**Background:**

The transition in 2015 to the Tenth Revision of the International Classification of Disease, Clinical Modification (ICD-10-CM) in the US led the Centers for Disease Control and Prevention (CDC) to propose a surveillance definition of traumatic brain injury (TBI) utilizing ICD-10-CM codes. The CDC’s proposed surveillance definition excludes “unspecified injury of the head,” previously included in the ICD-9-CM TBI surveillance definition. The study purpose was to evaluate the impact of the TBI surveillance definition change on monthly rates of TBI-related emergency department (ED) visits in Colorado from 2012 to 2017.

**Results:**

The monthly rate of TBI-related ED visits was 55.6 visits per 100,000 persons in January 2012. This rate in the transition month to ICD-10-CM (October 2015) decreased by 41 visits per 100,000 persons (*p*-value < 0.0001), compared to September 2015, and remained low through December 2017, due to the exclusion of “unspecified injury of head” (ICD-10-CM code S09.90) in the proposed TBI definition. The average increase in the rate was 0.33 visits per month (*p* < 0.01) prior to October 2015, and 0.04 visits after. When S09.90 was included in the model, the monthly TBI rate in Colorado remained smooth from ICD-9-CM to ICD-10-CM and the transition was no longer significant (*p* = 0.97).

**Conclusion:**

The reduction in the monthly TBI-related ED visit rate resulted from the CDC TBI surveillance definition excluding unspecified head injury, not necessarily the coding transition itself. Public health practitioners should be aware that the definition change could lead to a drastic reduction in the magnitude and trend of TBI-related ED visits, which could affect decisions regarding the allocation of TBI resources. This study highlights a challenge in creating a standardized set of TBI ICD-10-CM codes for public health surveillance that provides comparable yet clinically relevant estimates that span the ICD transition.

## Background

Traumatic Brain Injury (TBI) is “caused by a bump, blow, jolt, or penetration to the head that disrupts normal function of the brain” (Faul et al. 2010). In Colorado, TBIs contributed to 13 percent of all injury-related emergency department (ED) visits from 2012-2014 based on health care billing data coded in the International Classification of Diseases, Ninth Revision, Clinical Modification (ICD-9-CM) (Colorado Department of Public Health 2015). On October 1, 2015 the US implemented the tenth revision, ICD-10-CM (National Center for Health Statistics 2015).

Epidemiologists at the CDC and other public health agencies in the US use ICD-coded health care administrative billing data for public health surveillance of TBI (CDC 2015; Marr and Coronado 2004). The CDC proposed definition for TBI surveillance using ICD-10-CM codes does not include S09.90 for “unspecified injury of the head” (Hedegaard et al. 2016). However, the ICD-9-CM TBI surveillance definition included 959.01 for “unspecified injury of head” (CDC 2015; CDC 2006; Faul et al. 2010; Marr and Coronado 2004; Taylor et al. 2017) even after the publication of the Barell matrix that excluded unspecified head injury as a TBI (Barell et al. 2002; The Israeli Center for Trauma and Emergency Medicine Research 2005). ICD-10-CM codes provide more detail regarding the clinical nature of an injury (National Center for Health Statistics 2015), and the transition to ICD-10-CM could provide CDC the opportunity to better align the TBI surveillance definition with the clinical diagnosis of TBI and the injury diagnosis framework for ICD-10-CM (Hedegaard et al. 2020).

To date, no studies have assessed the effects of the transition on TBI-related ED visits and the inclusion/exclusion of unspecified head injury in the surveillance definition on trends spanning the coding transition. Studies assessed the positive predictive values (PPV) of the ICD-10-CM codes for TBI and unspecified head injury treated in the EDs or the PPV of TBI codes among hospitalizations but did not assess the impact of the proposed change in the surveillance definition on the magnitude and trend in TBI rates (Gabella et al. 2021; Peterson et al. 2021; Warwick et al. 2020). Studies have assessed changes in TBI ED rates during the ICD-9-CM era, but have not assessed trends for a period covering the transition to ICD-10-CM as well (Hsia et al. 2018; Taylor et al. 2017). One study of the transition to ICD-10-CM using interrupted time series focused on TBI-related hospitalizations among a subpopulation of adults aged 19–44 years in 2011–2017 where the primary reason (principal diagnosis) for the hospitalization was any traumatic injury (Sebastião et al. 2021).

The purpose of the study is to assess the impact of the transition to ICD-10-CM and the TBI definition change, specifically the exclusion of the code S09.90, on TBI-related ED visit trends among all ages from 2012 to 2017 in Colorado. The study is intended to raise awareness about the transition to ICD-10-CM to assure accurate interpretation of TBI trends using new coding guidelines (Slavova et al. 2018; Sebastião et al. 2021).

## Methods

The study team analyzed administrative billing data on ED visits for Colorado residents treated and released in Colorado non-federal, acute care hospitals from January 1, 2012 through December 31, 2017. At the time of the analysis, the data available were through 2017. This period provided more than 8 data points (rates) before and after the transition to ICD-10-CM, thereby providing enough power to detect change (Penfold and Zhang 2013). This period was long enough to account for seasonality in TBI-related ED visits.

The administrative billing records for ED visits contain standard information that hospitals must submit to private and government health insurance programs for reimbursement of treatment or to patients to pay, if they do not have health insurance (National Uniform Billing Committee 2021). Study staff searched all 30 discharge diagnoses fields on each billing record for any of the TBI codes. The ICD-9-CM codes used to identify TBI were: 800.0–801.9 (vault or basilar skull fracture), 803.0–804.9 (other skull fractures), 850.0–854.1 (intracranial injury, including concussion), 950.1–950.3 (injury to optic chiasm; optic pathways; visual cortex), 995.55 (shaken baby syndrome), and 959.01 (head injury, unspecified) (Marr and Coronado 2004). The ICD-10-CM codes proposed by CDC to identify TBI were: S02.0, S02.1- (fracture of skull), S02.8 (Fracture of other specified skull and fracture bones), S02.91 (unspecified fracture of skull), S04.02, S04.03-, S04.04- (injury to optic chiasm; optic tract and pathways; visual cortex), S06- (intracranial injury), S07.1 (crushing injury of skull), and T74.4 (shaken infant syndrome) but not code S09.90 for “unspecified injury of head” (Hedegaard et al. 2016).

A concept unique to the ICD-10-CM is the type of encounter (for active treatment, planned procedures in the recovery phase, or treatment of a sequela) indicated by seventh character in the codes. For greater comparability with ICD-9-CM selection criteria, excluded were ED billing records with only a proposed TBI ICD-10-CM code with a seventh character of “S”, indicating sequelae of TBI (Hedegaard et al. 2016). Not using ICD-9-CM codes for late effects and excluding records with ICD-10-CM codes indicating sequelae does not mean that the resulting TBI-related ED visits reflect visits for incident cases of TBI.

The outcomes were the monthly rates of TBI-related ED visits among the study population. Monthly rates were computed as a function of the total number of ED visits with a TBI code in each month. Specifically, the numerator for a rate was the monthly number of TBI-related ED visits. The denominators used to compute the monthly rate of TBI per 100,000 persons were annual state population estimates from the Colorado Department of Local Affairs (2018). The billing data did not contain an identifier to track individuals with multiple visits in a month or with multiple TBIs in a month, so the rates reflect rates of visits, not patients.

Trends in crude monthly rate of TBI-related ED visits were assessed with two regression models. The first model used rates based on the commonly used ICD-9-CM definition (that includes unspecified head injury) and the proposed CDC TBI surveillance definition that excludes unspecified head injuries after Oct 1, 2015. The second model used the same monthly rates for the ICD-9-CM era and for the ICD-10-CM era, used re-calculated rates with counts of unspecified injury of head (ICD-10-CM code S09.90) added to the numerator of TBI-related ED visits.

The statistical model was a segmented regression using an interrupted time series design based on a detailed methodology in Slavova et al. (2018) The analysis was conducted with SAS 9.4© 2018, using the SAS function PROC AUTOREG to estimate autoregressive parameters with a BACKSTEP option to select the correct order of parameters in the autoregressive error model. An alpha level of 0.05 was used to determine significance. The following regression model was used for analysis. Table 1 explains what each variable in the regression model means.

$$ {\mathrm{Y}}_{\mathrm{t}}={\upbeta}_0+{\upbeta}_1\ast {\mathrm{t}\mathrm{ime}}_{\mathrm{t}}+{\upbeta}_2\ast \mathrm{ICD}1{0\mathrm{CM}}_{\mathrm{t}}+{\upbeta}_3\ast \mathrm{time}\_\mathrm{after}\_\mathrm{ICD}1{0\mathrm{CM}}_{\mathrm{t}}+{\mathrm{v}}_{\mathrm{t}} $$

**Table 1:** Variables in the Interrupted Time Series equation^a^

Variable	Description
Y_t_	The rate of TBI related ED visits per 100,000 population in a given month (t).
time_t_	Represents month values from 1 (January 2012) to 72 (December 2017).
β_0_	The intercept or the outcome at the start of the ICD-9-CM period (baseline level at time 0, t = 0, of December 2011)
β_1_	The coefficient for the independent variable of time, that starts at 1 for the first month and 72 for the last month for the 72 total months in the six-year period from January 2012 to December 2017
β_2_	The coefficient indicating the level of change in the rate of TBI-related ED visits immediately after the transition
*ICD10CM*	A dummy variable indicating the start of ICD-10-CM (the interruption in the time series) is 0 for each month prior to October 2015, and 1 for each month beginning October 2015 and after.
β_3_	The coefficient indicates the change in the slope of the monthly TBI rate after the transition to ICD-10-CM, compared to slope *β*_*1*_
*time_after_ICD10CM*	The variable indicates in rank order or sequential order the months at and after the beginning of ICD-10-CM. This variable is 0 prior to ICD-10-CM, 1 for October 2015, 2 for November 2015, 3 for December 2015, etc.
*v*_*t*_	The error term represents the random variability not explained by the variables in model

^a^Slavova et al. (2018) provides further explanation of the variables

## Results

At the beginning of the study period (January 2012), the monthly rate of TBI-related ED visits per 100,000 persons was 55.6 (*p* < .0001) with an average increase of 0.33 TBI-related ED visits per month (*p* < .0001). By the end of the ICD-9-CM coding era (September 2015), the monthly rate had increased to 70.3 (*p* < .0001).

**Fig. 1:**
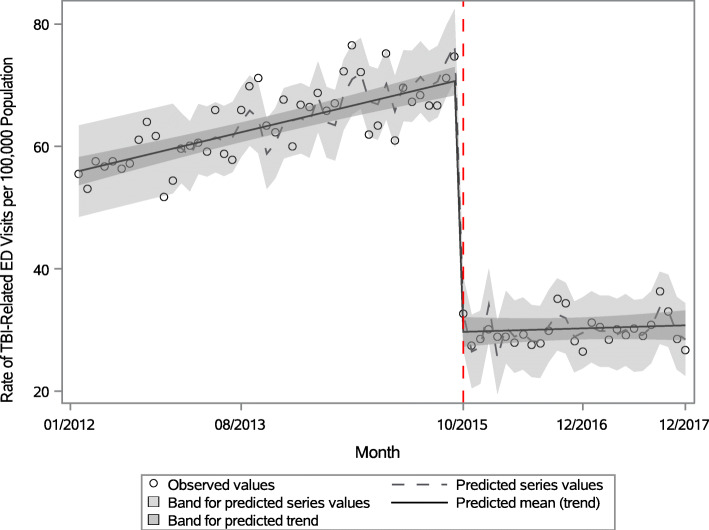
Monthly rates of TBI-related emergency department visits per 100,000 Colorado residents, 2012-2017^a^. ^a^ Figure 1 Footnote: Red dotted line on October 1, 2015 indicates the start of ICD-10-CM in the United States. Traumatic brain injury (TBI) estimates after the transition to ICD-10-CM (marked by red dotted line) do not include unspecified injury of head

From the first estimation model where TBI included unspecified head injury before but not after the transition to ICD-10-CM, the monthly rate in the first month of the ICD-10-CM era (October 2015) was 29.3, a decrease of 41 visits per 100,000 (*p* < .0001) from the prior month. After the ICD transition, the average monthly rate increase from October 2015 through December 2017 was 0.04 (Fig. 1), a decrease of 0.29 per 100,000 (*p* = 0.0004) from the ICD-9-CM era. This autoregressive model detected and accounted for positive serial correlation of the error terms every 12 months.

**Fig. 2:**
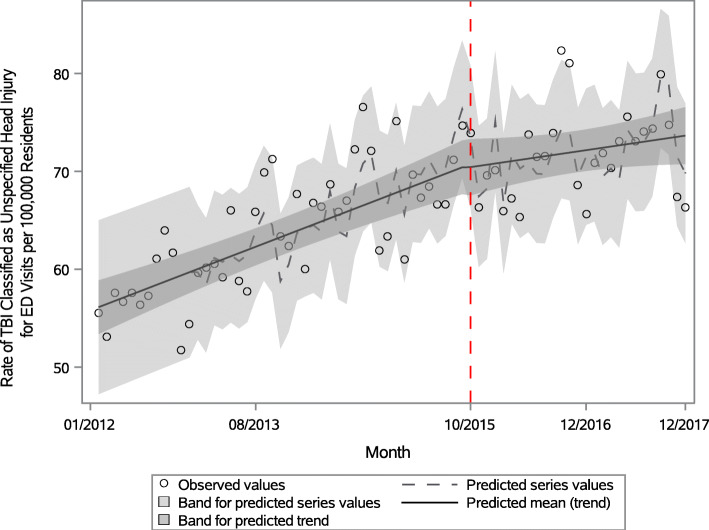
Monthly rates of TBI-related Emergency Department Visits per 100,000 Colorado Residents (with S09.90), 2012-2017^b^. ^b^Figure 2 Footnote: Red dotted line on October 1, 2015 indicates the start of ICD-10-CM in the United States. Traumatic brain injury estimates after the transition to ICD-10-CM (marked by red dotted line) include unspecified injury of head (S09.90)

Using the second estimation model, with inclusion of ICD-10-CM code S09.90, the transition to ICD-10-CM in October 2015 was not significant (*p* = 0.94) and resulted in a smooth trend over time (Fig. 2). The average monthly rate from October 2015 through December 2017 increased by 0.12, a decrease by 0.20 per 100,000 persons (*p* = 0.04) from the ICD-9-CM era.

## Discussion

Our findings suggest that the exclusion of code S09.90 contributed to the immediate decrease of 41 visits per 100,000 persons in the monthly rate of TBI-related ED visits in October 2015. This decrease represented over half of the monthly rate in September 2015. The proportion of TBI-related ED visits with 959.01 from 2012 through 2015 was constant, and therefore, does not explain the higher average increase in the monthly rate during the ICD-9-CM era compared to the ICD-10-CM era (0.33 vs. 0.12 from the first model). Unspecified head injuries (code 959.01) represented 64% of the TBI-related ED visits in 2012, 62% in 2013 and 2014, and 62% in the first 9 months of 2015. Structural changes in specific ICD-10-CM codes for TBI, compared to ICD-9-CM codes, appear to have expanded the capture of TBI post transition, including potentially false-positive TBI (Sebastião et al. 2021). These structural code changes might have contributed to the remaining increase in average monthly rates post transition, even after including unspecified head injury (the second model).

Additionally, the upward trend in TBI-related ED visits during both ICD-CM eras could be attributed to many factors: willingness to seek medical care and visit an ED, increased health insurance coverage, older adults with a higher incidence of TBI among an aging population, and/or increased incidence of TBI (Hsia et al. 2018; Nikpay et al. 2017). Reporting bias due to shifting care from EDs to urgent care or outpatient settings during the ICD-10-CM period seems an unlikely alternative explanation of the study findings, unless such shifting occurred differently for patients treated and released from EDs with TBI diagnoses than for patients diagnosed with unspecified head injury. However, some of the increase could reflect increase in ED capacity over this time period. Overall, Colorado ED visits with any diagnosis increased 8.7% from 2013 to 2017, and the number of acute care hospitals and general licensed hospitals with EDs in Colorado increased 7.4% from 81 in 2012 to 87 in 2017.

This study also found seasonality. The seasonality or correlation every 12 months in monthly TBI-related ED visits could result from the seasonality in the mechanism of injury, which another state documented (Slavova et al. 2018). As an example, the snow conditions every January in Colorado could be similar and could affect driving conditions that increase motor-vehicle related TBIs. January is also ski season in Colorado. Summer months could bring increased recreational activities, such as hiking, biking, amateur sports, and vacation driving.

Study limitations include not assessing other factors that could have influenced monthly TBI-related ED visit rates such as legal changes and increased awareness of brain injury among the public (National Conference of State Legislators 2018; CDC 2015). The study results are not necessarily representative of other states or countries. This study did not assess whether training or other preparation for the ICD-10-CM transition influenced physician medical record documentation or the accuracy of billing diagnosis coding. The study did not cluster data by hospital or account for differences in coding or billing practices by hospital, potentially resulting in a type-1 error inflation. Finally, ICD-CM codes are designed for billing purposes and have imperfect sensitivity and specificity when used for surveillance purposes.

The monthly TBI rate between 2012 and 2015 may overestimate the true TBI rate. A validation study of the TBI ICD-9-CM definition in ICD-9-CM in the ED at a large urban hospital in 2003 found that only 20% of cases coded with 959.01 for unspecified head injury met the clinical definition of TBI (Table 3 in Bazarian et al. 2006). Additionally, the Barell Matrix, utilized by CDC, does not include the ICD-9-CM code 959.01 in the TBI definition and considers these injuries “other head, face and neck” (Barell et al. 2002; The Israeli Center for Trauma and Emergency Medicine Research 2005).

A strength of this study is that it was a large statewide study with numerous acute care hospitals, and not limited to one hospital or one hospital system. The regression models accounted for seasonality, corrected the regression estimates for the positive autocorrelation of the error terms, and assessed any changes in magnitude and trend of the monthly rates of ED visits per 100,000 population due to including or excluding unspecified head injuries in the TBI surveillance definition.

## Conclusions

This study shows the importance of understanding the diagnosis codes used to define monthly rates of TBI-related ED visits. The drastic reduction in the trend of TBI estimates after October 1, 2015 using ICD-10-CM codes, could affect decisions regarding the allocation of TBI resources. Public health practitioners should be aware that this interruption is primarily due to the change in the TBI surveillance definition and not the coding transition itself or its limitations.

This study highlights a challenge in creating a standardized set of TBI ICD-10-CM codes for public health surveillance that provides comparable yet clinically relevant estimates over time. These study results based on 6 years of ED billing data from a single state can help inform the next steps for finalizing a public health surveillance definition of TBI until the next major change in the ICD-CM coding scheme, and this study should be repeated in other states and jurisdictions.

## Data Availability

The datasets used and/or analyzed during the current study are available from the corresponding author on reasonable request.
